# Unexplained Fever in Infancy: Report of a Rare Case of Hypohidrotic Ectodermal Dysplasia in an Infant

**DOI:** 10.7759/cureus.39489

**Published:** 2023-05-25

**Authors:** Zainab S Gilitwala, Shalmali R Satpute

**Affiliations:** 1 Pediatrics, Rajarshee Chhatrapati Shahu Maharaj Government Medical College, Kolhapur, IND

**Keywords:** hypohidrotic ectodermal dysplasia, hyperpyrexia, hypodontia, hypohidrosis, hypotrichosis

## Abstract

Hypohidrotic ectodermal dysplasia (HED) is a genetic condition that affects structures derived from the ectoderm during embryonic development. These structures include the outermost layer of the primary germ layers, which give rise to various body parts such as the ears, eyes, lips, and mucous membranes of the nose and mouth. Due to the impact on these structures, hypohidrotic ectodermal dysplasia can manifest differently in various age groups. However, the three primary characteristics typically associated with this condition are hypotrichosis, hypohidrosis, and hypodontia or anodontia. Here, we present a case of a male infant, aged 2 months, who was brought to our attention due to symptoms of unexplained fever and irritability. The child's family history was noteworthy, as an older sibling had distinctive features of ectodermal dysplasia. This information led us to consider the possibility of this diagnosis. This case report aims to highlight the distinctive features of such cases that facilitate the identification of this condition and its related complications. By sharing this case, we intend to raise awareness and encourage timely detection, diagnosis, and proper treatment of patients with this condition.

## Introduction

Ectodermal dysplasia is a rare inherited condition with 1:10,000 to 1:100,000 prevalence worldwide that impacts structures derived from the ectoderm, such as nails, hair, teeth, and sweat glands. The most prevalent form of this disorder is hypohidrotic and hidrotic ectodermal dysplasia, with oligodontia being the most prominent dental characteristic [1]. Hypohidrotic ectodermal dysplasia is identified by reduced or absent sweat glands leading to hypohidrosis, sparse and light-colored scalp and body hair, known as hypotrichosis, and hypodontia. This condition also presents other noticeable features such as a significant forehead, a nose with a flattened bridge, and excessive pigmentation around the eyes or periorbital hyperpigmentation [2,3]. The dental symptoms comprise partial or complete absence of teeth or anodontia, teeth with an abnormal shape, enamel hypoplasia, reduced height of the alveolar ridge, maxillary retrusion, and an elevated arch on the palate [1]. In this case report, we are showcasing two siblings who exhibited the distinctive traits of hypohidrotic ectodermal dysplasia. We present a case of an infant with non-specific symptoms of pyrexia and irritability. The child was misdiagnosed as meningitis, but it was the striking family history and astounding clinical picture of the older sibling that led to the diagnosis of hypohidrotic ectodermal dysplasia in the infant.

## Case presentation

**Figure 1:**
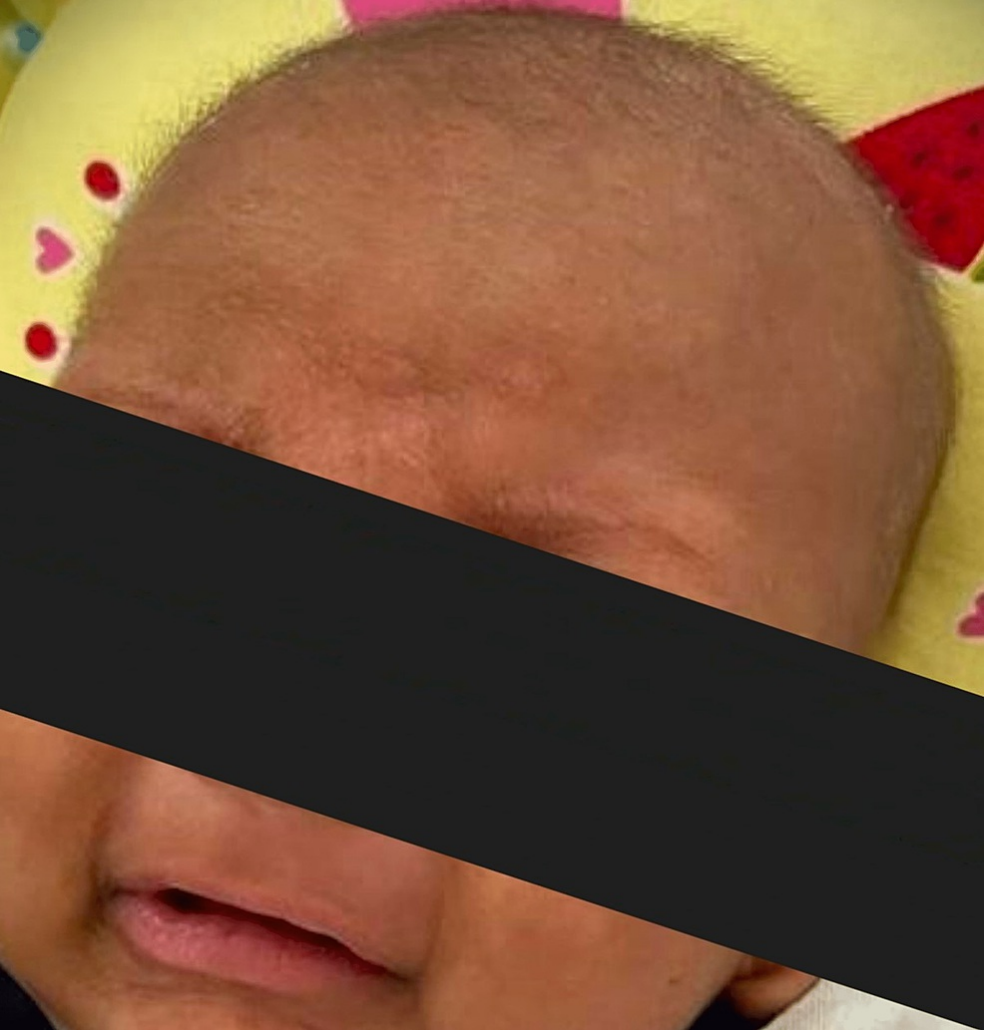
Infant with no eyebrows and sparse, hypopigmented, thin hair

A 2-month-old male child presented to us with high-grade fever and irritability for 4 days. The infant also was lethargic and refused to feed. Moreover, a significant history of the infant not sweating since birth was noted. There was no history of seizures, loose stools, or vomiting. Prior to visiting our hospital, the infant was evaluated for meningitis and received some oral antibiotics. The birth history of the infant was unremarkable, while their family history was quite striking. The child was the fifth in birth order, born to parents who were third-degree consanguineous partners. One of the female siblings had previously passed away at the age of two months, and the cause of death was unknown to the parents, although there was a history of unexplained fever prior to the sudden death. The child was immunized up to date and had no prior illness. On examination, the infant had no eyebrows and had very sparse, hypopigmented, thin hair as seen in Figure 1.

**Figure 2:**
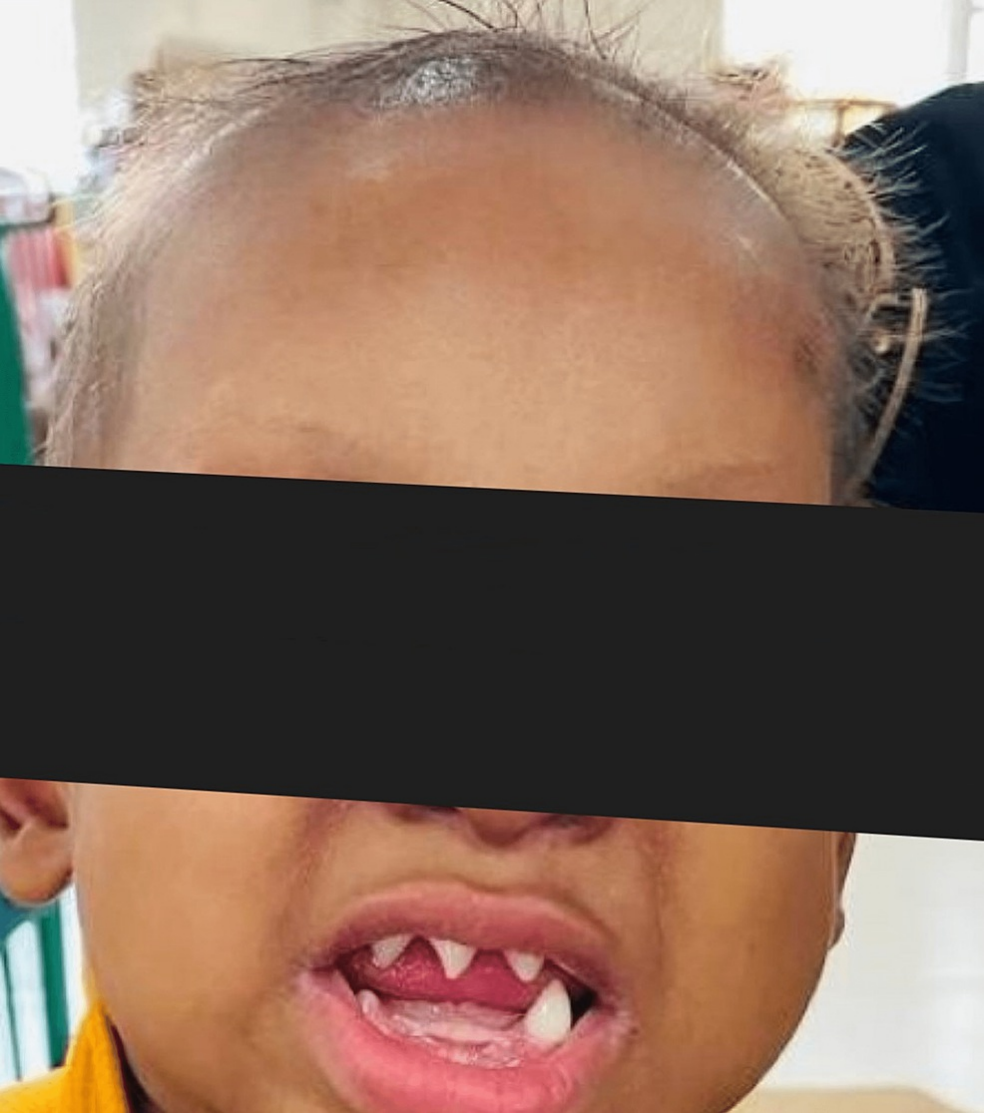
The patient's 5-year-old sibling presenting with an absence of eyebrows, alopecia, missing teeth, and a few conical teeth.

The infant had a continuous high-grade fever of 101 °F. The fever had no diurnal variation. The temperature of the patient did not reach baseline even after treatment with antipyretics. His skin was warm and dry. Sparse eyelashes were noted on both upper and lower eyelids, and a depressed nasal bridge was observed. The examination of the nervous system was within normal limits. No other foci of infection were found on other systemic examinations. The patient's complete blood count, urine analysis reports, and cerebrospinal fluid examination were all normal and did not indicate the presence of any infection. A comprehensive metabolic panel also revealed no abnormality. A brain MRI revealed no abnormalities, ruling out the presence of meningitis. Additionally, an ultrasound examination of the abdomen and pelvis did not indicate the presence of any deep-seated abscess. Similarly, the ultrasound of both hips and knees did not show any signs of arthritis. It was the striking family history and the mild dysmorphism that led to further evaluation of the family members. The elder female sibling, who was 5 years old, had no eyebrows, alopecia, missing teeth and a few conical teeth, periorbital hyperpigmentation, and sparse light hair over the scalp with a classical history of anhidrosis, as seen in Figure 2.

These characteristic findings in the family members with similar clinical features in the patient led to the diagnosis of hypohidrotic ectodermal dysplasia. To confirm the diagnosis of the condition, genetic testing or a skin biopsy is usually recommended. However, in this case, neither test was performed as the patient's parents did not provide consent. The parents received education about the condition and were advised to take supportive measures, such as preventing the child from exposure to hot environments. They were also recommended to use light cotton clothing and cotton bed linen. Given the hereditary nature of the condition and the history of consanguinity, the patient's siblings were also assessed. The parents were advised to undergo genetic counseling and prenatal testing in preparation for future pregnancies.

## Discussion

Hypohidrotic ectodermal dysplasia (HED) is a genetic disorder that encompasses a complex set of inherited conditions. This disorder is identified by the impaired development of two or more anatomical structures derived from the ectoderm, such as the skin, hair, nails, teeth, and sweat glands [4]. While there are autosomal recessive and dominant forms, X-linked (XL) is the most prevalent type of the disorder. This X-linked HED phenotype is linked to mutations in the gene responsible for encoding the transmembrane protein ectodysplasin-1 (EDA1). This protein facilitates signal transduction from the ectoderm to mesenchyme during fetal development, resulting in the formation of structures derived from the ectoderm [5]. The most common phenotype is hypohidrotic/anhidrotic ectodermal dysplasia, also known as Christ-Siemens-Touraine syndrome. It is an X-linked disorder characterized by heat intolerance, absence of sweat glands, and abnormal spiky or absent teeth. Depending upon the physical features of the affected individual, HED can be diagnosed after infancy in most cases. As a result of impaired thermal perception, patients experience overheating (hyperthermia), particularly during the summer.

The abnormal dentition includes an incorrect number and shape of teeth, which can lead to difficulties in speech, impaired mastication, and aesthetic discomfort. Abnormalities in the number of teeth are frequently observed. The shape of the teeth is frequently conical, bulbous, or taurodontic, and they are widely spaced, without any points of contact. The weakened enamel is vulnerable to decay and physical harm [6]. The teeth may not erupt properly, and the total number of teeth can be significantly reduced. HED can also cause atrophic inflammation of the mucosa in the oral cavity and throat, resulting in a hoarse voice and difficulties swallowing. The skin is often thin, delicate, and dry, with inadequate pigmentation, except for the areas around the eyes and mouth, where it appears wrinkled and hyperpigmented, leading to a prematurely aged appearance [7-9].

Patients with ectodermal dysplasia (ED)who have an immunodeficiency component may experience hypogammaglobulinemia, which can lead to impaired lymphocyte proliferation and compromised cell-mediated immunity. Radiographic imaging of the hands, feet, or both may reveal distinct skeletal abnormalities. Sweat pore counts, pilocarpine iontophoresis and skin biopsies can help diagnose hypohidrosis and a decrease in the number of eccrine glands. Relatives at risk should be offered molecular genetic testing if the pathogenic variant(s) specific to their family are known, to enable early diagnosis and treatment, particularly to prevent hyperthermia. If a woman has a known carrier status of X-linked HED, prenatal diagnosis of the condition can be determined through molecular genetic testing after amniocentesis or chorionic villus sampling. Tooth germ sonography is another non-invasive option for diagnosing HED prenatally [10-11 ]. In our case, we arrived at the diagnosis primarily through radiographs, family history, and our own clinical examination.

The main objective of treating a patient with ectodermal dysplasia is to improve their appearance, and it typically involves a collaborative team approach that includes pediatricians, pediatric dentists, prosthodontists, dermatologists, otolaryngologists, speech therapists, and psychologists [2]. Early dental intervention is crucial for patients with EDto prevent complications and maintain the alveolar ridge for future dental treatments. Prosthetic teeth can improve mastication and speech, while aesthetic dental interventions can enhance self-image and overall oral health in patients with malformed teeth and malocclusion. Complete denture prosthodontics can also be performed in children with ED[12]. One of the major complications of this condition is hyperpyrexia in infancy, which can cause sudden infant death [13].

HED has no definitive cure, and its management is focused on alleviating associated symptoms. Patients are advised to stay in cool environments with air conditioning, drink cool liquids frequently, and wear lightweight and breathable clothing. Artificial tears may be used to protect the cornea [4]. Surgical intervention to repair cleft palate can improve speech and facial deformities.

Our case study has certain limitations. Since genetic testing or skin biopsy was not performed, we could not determine the exact gene mutation to confirm the diagnosis. The patient received supportive care and was referred to a pediatric dermatologist and dentist for further follow-up.

## Conclusions

Hypohidrotic ectodermal dysplasia is an uncommon condition that leads to hyperpyrexia in infancy, which can cause sudden infant death. Early diagnosis and awareness of this benign condition are crucial as the classical features of this condition are not prominent during early infancy, hence easily missed by physicians. While no curative treatment exists for this condition, patients can be effectively managed through a multidisciplinary approach that involves a team of healthcare professionals, including pediatricians, expert dentists, and dermatologists, who provide supportive care. As hypohidrotic ectodermal dysplasia is an inherited condition, parents can benefit from genetic counseling and prenatal diagnosis to assist them with future pregnancies.
